# SIRT1 and thrombosis

**DOI:** 10.3389/fmolb.2023.1325002

**Published:** 2024-01-18

**Authors:** Alessandra Bettiol, Maria Letizia Urban, Giacomo Emmi, Silvia Galora, Flavia Rita Argento, Eleonora Fini, Serena Borghi, Giacomo Bagni, Irene Mattioli, Domenico Prisco, Claudia Fiorillo, Matteo Becatti

**Affiliations:** ^1^ Department of Experimental and Clinical Medicine, University of Firenze, Firenze, Italy; ^2^ Department of Experimental and Clinical Biomedical Sciences “Mario Serio”, University of Firenze, Firenze, Italy

**Keywords:** SIRT1, thrombosis, oxidative stress, atherosclerosis, sirtuins

## Abstract

Thrombosis is a major cause of morbidity and mortality worldwide, with a complex and multifactorial pathogenesis. Recent studies have shown that SIRT1, a member of the sirtuin family of NAD + -dependent deacetylases, plays a crucial role in regulating thrombosis, modulating key pathways including endothelial activation, platelet aggregation, and coagulation. Furthermore, SIRT1 displays anti-inflammatory activity both *in vitro*, *in vivo* and in clinical studies, particularly via the reduction of oxidative stress. On these bases, several studies have investigated the therapeutic potential of targeting SIRT1 for the prevention of thrombosis. This review provides a comprehensive and critical overview of the main preclinical and clinical studies and of the current understanding of the role of SIRT1 in thrombosis.

## 1 Introduction

Sirtuins (Silent Information Regulator-SIRT proteins), also known as Sir2 family proteins, are a class III histone deacetylase enzyme that utilize nicotinamide adenine dinucleotide (NAD+) as a coenzyme to deacetylate lysine residues in both histone and non-histone proteins (Kupis et al., 2016). Recent research has revealed that sirtuins, in addition to deacetylation, catalyze also other enzymatic reactions, including demalonylation, desuccinylation, demyristoylation, and mono-adenosine diphosphate (ADP)-ribosylation (Dai et al., 2018).

The yeast and mammalian SIRT1 families responsible for cellular regulation consist of 7 members, namely, SIRT1‐7, classified into four groups: SIRT1‐3 in class I, SIRT4 in class II, SIRT5 in class III, and SIRT6‐7 in class IV (Michan and Sinclair, 2007). Mammalian sirtuins can be grouped based on their subcellular localization, with SIRT1, SIRT6, and SIRT7 located in the nucleus, and SIRT3, SIRT4, and SIRT5 in the mitochondria, while SIRT2 is predominantly expressed in the cytoplasm (Houtkooper et al., 2012). The various subcellular locations, expression patterns, and substrates of sirtuins are closely linked to their biological functions (Bai and Zhang, 2016). Thanks to their capability of targeting post-translational acyl modifications of different cellular substrates, sirtuins are a critical component in numerous biological pathways, including mitochondrial energetic metabolism, cellular homeostasis and proliferation, DNA repair, and antioxidant activity (Mei et al., 2016; Lee, 2019). Furthermore, *in vitro* studies have shown that sirtuins play a role in aging, transcription, apoptosis, inflammation, and cellular stress resistance (Vachharajani et al., 2016).

In this review, we will discuss the biological functions of SIRT1, focusing on its role in thrombo-inflammation and thrombosis. The potential clinical use of sirtuins-modulating agents for thrombosis prevention will be also discussed.

## 2 SIRT1: molecular and functional features

The yeast Sir2 protein contains a domain comprising approximately 275 amino acid residues that is conserved in Sir2-like proteins found in both prokaryotes and eukaryotes (Brachmann et al., 1995). SIRT1, the most extensively studied and characterized sirtuin, is highly homologous to basic members of the Sir2 family in yeast (32.44 Percent Identity Matrix by Clustal 2.1) (Wang et al., 2021). The human SIRT1 gene, located at 10q21.3 ^11^, contains a 33,715 bp region, which is highly conserved and comprises nine exons encoding a 747 amino acid protein. Like the other mammal sirtuins, SIRT1 has highly conserved NAD-dependent sirtuins core domain, first identified in the founding yeast SIR2 protein; also, the human SIRT1 protein contains a potential nuclear localization signal (KRKKRK) in amino acids 41–46, which is a known intranuclear localization of the yeast Sir2p (Alqarni et al., 2021).

This protein includes an N-terminal nuclear localization signal and a conserved 275 amino acid catalytic core. The catalytic core, defined as an NAD+/NADH binding protein (Finkel et al., 2009), is a large Rossmann fold domain with a small zinc finger domain, in which the Zn^2+^ is tetrahedrally coordinated by the thiols of four cysteine residues [Cys–(X)2–Cys–(X)15–20–Cys–(X)2–Cys]. This motif is highly conserved in most sirtuin family members. The Zn^2+^ is not directly involved in the catalysis, but it forms a zinc-ribbon motif keeping a cleft between the zinc-ribbon and the large domain of SIRT1, thus being essential for SIRT1 activity (Finkel et al., 2009).

The NAD binding sites are located at the junction of the two domains, and the acetylated substrate is bound to the end of the gap near a NAD^+^ glycosylation site. Thanks to this peculiar structure, SIRT1 is involved in multiple cellular pathways, such as gene transcription, DNA repair and replication, and metabolic regulation. SIRT1 deacetylates histones and many non-histone proteins, modulating the electrostatic properties of DNA–histone interaction and ultimately regulating transcription (Alves-Fernandes and Jasiulionis, 2019). Notably, SIRT1 does not directly bind DNA, but is recruited by various chromatin-associated factors to their binding sites, where it acts a coordinator in the formation of both facultative and constitutive heterochromatin. SIRT1 deacetylates lysine residues of the N-terminal tails of H3 and H4, (preferentially H4K16, and to a lower rate H3K9, H3K14, H4K8, and H4K12), as well as the linker histone H1 at Lys26 (H1K26) (Vaquero et al., 2004).

Additionally, several non-histone SIRT1 protein targets have been observed and studied (Yuan et al., 2007; Hughes et al., 2011; Jiao and Gong, 2020), including tumor protein 53 (p53). p53 is a potent tumor suppressor and was the first non-histone deacetylation target identified for SIRT1. SIRT1 regulates both p53 transcription-dependent and transcription-independent apoptosis: it binds deacetylates p53 at K379 (human K382) and blocks p53 nuclear translocation. Deacetylated p53 translocates onto the mitochondrial outer-membrane and releases proapototic BCL protein BAX, which leads to the release of cytochrome c from the mitochondria and initiates p53 transcription-independent apoptosis (Yi and Luo, 2010).

Also, SIRT1 interacts with forkhead transcription factor O (FoxO) proteins, which physiologically regulate apoptosis, cell cycle arrest, differentiation, activation of genes for DNA repair and resistance to oxidative stress. By deacetylating FoxO3 and/or FoxO4, SIRT1 mediates an attenuation of FOXO-induced apoptosis and a potentiation of FoxO-induced cell-cycle arrest, thus being crucial for the biology of aging (Giannakou and Partridge, 2004).

Among non-histone targets of SIRT1 is also peroxisome proliferator-activated receptor-γ co-activator-1α (PGC-1α), which is a nuclear transcriptional co-activator of nuclear receptors and other transcription factors and a regulator of mitochondrial biogenesis (Shi and Gibson, 2007). The deactylation of PGC-1α by SIRT1 enhances its activity, promotes mitochondrial biogenesis, and plays a protective role in neuronal injuries and in ischemic heart disease (Zhou et al., 2018).

Moreover, an antagonistic crosstalk exists between SIRT1 and nuclear factor κB (NF-κB), a molecular that glycolytic energy flux during inflammatory responses. By deacetylating the p65 subunit of NF-κB complex, SIRT1 inhibits NF-κB signaling, enhances oxidative metabolism [also via the activation of 5′ adenosine monophosphate-activated protein kinase (AMPK), peroxisome proliferator-activated receptor alpha (PPARα) and PGC-1α] and promotes the resolution of inflammation. On the other hand, NF-κB downregulates SIRT1 activity through the expression of miR-34a, IFNγ, and reactive oxygen species, stimulating inflammatory responses as reported in many chronic metabolic and age-related disorders (Kauppinen et al., 2013).

The subcellular localization of SIRT1 differs among various cell types. In young mice, SIRT1 is expressed in the nuclei of cardiomyocytes, while in adult mice, it is expressed in both nuclei and cytoplasm (Guarente, 2007). Nuclear SIRT1 was observed in models of heart failure, such as post-myocardial infarction in rats and dilated cardiomyopathy in humans (Tanno et al., 2010). SIRT1 indeed regulates endothelium angiogenic regulation during vascular growth (Wang et al., 2019), and knockout mice developed abnormal cardiac functions. Also, SIRT1 has anti-apoptotic actions in cardiomyocytes, counteracts endoplasmic reticulum stress, enhances myocardial contractility and resistance to ischemia/reperfusion damage. Indeed, SIRT1 is upregulated during pressure overload, caloric reduction, and physical exercise, and is downregulated in acute ischemia. Accordingly, constitutional overexpression of in transgenic mice overexpressing SIRT1 had a reduced contractile function of the myocardium and, a U-shaped dose-response curve was suggested linking SIRT1 to myocardial function (Ministrini et al., 2021).

**FIGURE 1:**
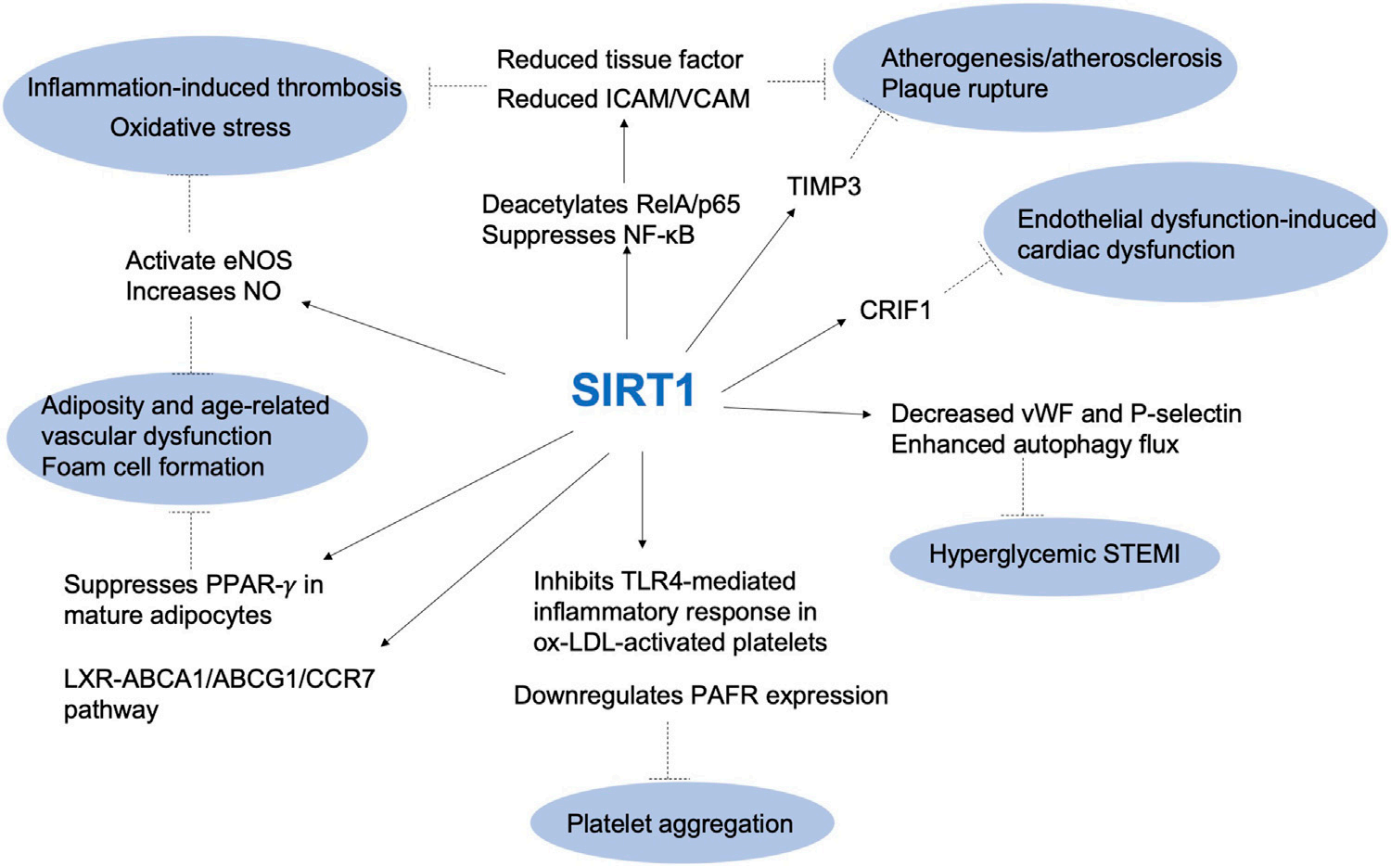
The role of SIRT1 in thrombotic pathways. ABCA1: ATP Binding Cassette Subfamily A Member 1; ABCG1: ATP binding cassette subfamily G member 1; CCR7: chemokine (C-C motif) receptor 7; CRIF1: CR6-interacting factor 1; eNOS: endothelial nitric oxide synthase; ICAM: intercellular adhesion molecule; LXR: liver X receptors; NF-κb: nuclear factor κb; NO: nitric oxide; Ox-LDL: oxidized low-density lipoprotein; PAFR: platelet-activating factor receptor; PPAR-γ: Peroxisome proliferator-activated receptor gamma; STEMI: ST elevation myocardial infarction; TIMP3: tissue metalloproteinase 3; TLR4: Toll-like receptor 4; VCAM: vascular cell adhesion molecule; vWF: von Willebrand factor.

Recent research has highlighted the role of SIRT1 in thrombotic pathways, particularly in relation to cardiovascular functions and vascular biology, such as inflammation, cholesterol transport, endothelial activation, platelet aggregation and foam cell formation, suggesting that this protein plays a key role in thromboinflammatory pathways (Soni et al., 2021) (Figure 1). Consequently, SIRT1 activation could be a promising therapeutic strategy for treating arterial thrombosis and atherosclerosis (Becatti et al., 2012; Ma and Li, 2015).

## 3 SIRT1 and endothelial cells

Endothelial cells are responsible for forming a single layer that lines the interior of blood vessels. They are critical for maintaining physiologic blood flow and controlling vascular hemostasis at sites of injury by facilitating various mechanisms, both procoagulant and anticoagulant. In normal conditions, endothelial cells act as a non-adhesive surface, preventing platelet activation and the initiation of the coagulation cascade. When there is damage to the blood vessel, platelets rapidly adhere to the exposed sub-endothelial extracellular matrix and form a clot, effectively sealing the injured vessel wall and preventing excessive blood loss. The mechanisms underlying endothelial cell activation and subsequent thrombosis are complex and multifaceted (Wang et al., 2018). Endothelial cell activation can be driven by various factors such as infection, inflammation, and mechanical injury. Recent studies have also highlighted the contribution of endothelial cells in thromboinflammation, a process that involves the interaction between thrombosis and inflammation (Neubauer and Zieger, 2022). Endothelial cells contribute to this process by releasing proinflammatory cytokines and chemokines, promoting leukocyte recruitment, and enhancing the activation of the coagulation cascade (Shao et al., 2020).

Endothelial SIRT1 plays an essential anti-atherosclerotic role by inhibiting endothelial apoptosis and promoting vascular endothelial function via nitric oxide (NO) synthase (eNOS) expression (Lee and Im, 2021). *In vitro*, SIRT1 reduces tissue factor expression by deacetylating RelA/p65 and suppressing NF-κB pathways, while *in vivo*, SIRT1 plays an anti-atherogenic role by suppressing NF-κB signaling (Zhang et al., 2009; Rothgiesser et al., 2010), which reduces aortic endothelial expression of intercellular adhesion molecule 1 (ICAM-1) and vascular cell adhesion molecule 1 (VCAM-1) (Stein et al., 2010).

Moreover, a recent study found that cilostazol, an antiplatelet drug, activated Krüppel-like Factor 2 (KLF2) expression and KLF2-related endothelial function, including eNOS activation, NO production, and endothelial thrombomodulin secretion in human umbilical vein endothelial cells (HUVECs) via SIRT1 activation (Wu et al., 2021a). These results suggested that the antithrombotic and vasculoprotective effects of cilostazol may be exerted trough SIRT1 signalling pathway.

The key role of SIRT1 pathway in thromboinflammation was also confirmed by Wu and collaborators that have investigated the role of the SIRT1/FoxO1 pathway in regulating autophagy and the release of von Willebrand factor (vWF) and P-selectin in HUVEC cells treated with oxidized low density lipoprotein (ox-LDL) (Wu et al., 2019). The results showed that ox-LDL increased vWF and P-selectin secretion and decreased the SIRT1/FoxO1 pathway, as well as increased the expression of autophagy-related proteins LC3-II/I and p62. Activation of the SIRT1/FoxO1 pathway with SIRT1 activators resveratrol and SRT1720 decreased the secretion of vWF and P-selectin, and enhanced autophagy flux through promotion of Rab7 expression (Wu et al., 2019).

In another study, D’Onofrio and co-workers showed that hyperglycemic thrombi had enhanced reactive oxygen species (ROS), pro-inflammatory/pro-thrombotic markers, as well as a lower expression of endothelial SIRT1. This study suggested that the SIRT1 pathway is involved in the enhanced pro-inflammatory and pro-thrombotic state of coronary thrombi in hyperglycemic ST elevation myocardial infarction (STEMI) patients (D Onofrio et al., 2020).

The role of SIRT1 regarding the relationship between hyperlipidemia and endothelial activation was also investigated. Yin and colleagues indeed generated a double gene knockout (KO) mice that were deficient of caspase-1 and ApoE (ApoE^−/−^/caspase-1−/−) demonstrating that caspase-1 activation significantly promotes endothelial activation, monocyte recruitment, and atherogenesis through SIRT1 pathway (Yin et al., 2015). From a mechanism perspective, ox-LDL are known to promote the activation of pro-caspase-1 to activated caspase-1, via an increased ROS concentration within the endothelial cell, and caspase-1 on its turn inactivates SIRT1. In this double KO mouse model, caspase-1 inhibition led to an accumulation of SIRT1 in the ApoE (−/−) aorta, and SIRT1 inhibited caspase-1 upregulated genes via activator protein-1 (AP-1) pathway.

More recently, the transgenic mouse model was used to investigate the impact of SIRT1 overexpression in endothelial dysfunction-induced cardiac dysfunction. In particular, the study focused on the role of CR6-interacting factor (CRIF1), which is essential for peptide synthesis and oxidative phosphorylation in mitochondria. The researchers used a mouse model to investigate the impact of endothelial cell-specific CRIF1 deletion on cardiac function and whether it was mediated by the antioxidant protein SIRT1. They found that mice with endothelial cell specific CRIF1 deletion had severe cardiac dysfunction, reduced ATP levels, inflammation, and oxidative stress in the cardiac tissues. Additionally, treatment with the SIRT1 activator SRT1720 improved cardiac function by activating eNOS, reducing oxidative stress, and inhibiting inflammation. The study indeed suggested that endothelial CRIF1 is critical for preserving cardiac function and that SIRT1 induction could be a promising therapeutic approach for endothelial dysfunction-induced cardiac dysfunction (Piao et al., 2021).

SIRT1 has also been found to play a crucial role in the development of obesity- and age-related vascular dysfunction. SIRT1 is often referred to as a ‘Master Metabolic Regulator’, as it regulates key energy processes in the liver, macrophage, brain, pancreas, hypothalamus and adipose tissue. This includes a regulation of gluconeogenesis, fat mobilization, lipogenesis and fatty acid oxidation [through interaction with key transcription factors such as target of rapamycin 2 (TORC2), PPARα, PGC-1α, sterol regulatory element binding protein (SREBP), liver X receptors (LXR), farnesoid X receptor (FXR)], insulin secretion, sense nutrient availability [via the hypothalamic deacetylation and activation of FoxO1, and thus inhibition of propiomelanocortin and increase of agouti-related protein (AgRP) expression, as well as via SIRT1 regulation of mTOR signaling], and a regulation of the activity of the circadian clock (via the modulation of CLOCK-BMAL1-mediated transcription and the deacetylation of PER, a negative regulator of CLOCK-BMAL1 transcription) (Schug and Li, 2011). Also, SIRT1 represses the PPARγ nuclear receptor, down-regulating adipocyte differentiation and maturation. In obesity, SIRT1 counteracts obesity-induced inflammation in macrophages by deacetylating the p65 subunit of the NF-κB transcription factor. Accordingly, SIRT1 reduction in macrophages is associated with an increased production and release of proinflammatory cytokines, ROS, and macrophage infiltration, promoting chronic inflammation and exacerbating visceral obesity (Campagna and Vignini, 2023).

Regarding aging, SIRT1 regulates several aging-related signaling pathways: this includes the inhibition of the p-65/NF-κB and of the p53 pathways, resulting in a reduced inflammation, DNA damage and oxidative stress; concomitantly, SIRT1 promotes the tuberous sclerosis protein 2 (TSC2)/mTOR, LKB1/AMPK, PGC-1α, FoxO pathways, with consequent anti-aging activities (Chen et al., 2020).

Accordingly, SIRT1 exerts protective effects against cardiovascular aging, through the inhibition of ROS production (via FoxOs and eNOS activation and p66Shc suppression), the suppression of inflammation (through reduction of NF-κB and AP-1), and the promotion of autophagy [by deacetylating FoxOs and autophagy-related genes (Atgs) 5,7 and 8, and negatively regulating mTOR] (Luo et al., 2014). During aging, the expression and activity of SIRT1 decline, leading to a reduction in the production of NO and an increase in ROS production at blood vessels level. These modifications can impair the function of the blood vessels and increase the risk of cardiovascular complications such as hypertension, atherosclerosis, and stroke (Kane and Sinclair, 2018).

A recent study specifically investigated the role of SIRT1 in age- and obesity-related microvascular dysfunction in humans. Ninety-five patients undergoing laparoscopic surgery were classified in four groups based on their body mass index (BMI) and age. Endothelial function was measured before and after incubation with the SIRT1 agonist SRT1720. The results showed that SIRT1 is directly involved in the earliest age- and obesity-induced microvascular damage. Through a complex epigenetic control mainly involving p66Shc and Arginase II, SIRT1 regulates the levels of mitochondrial ROS and NO, and the expression of key proteins from the mitochondria respiratory chain, subsequently restoring endothelial dysfunction (Mengozzi et al., 2022). Authors suggested that early targeting of SIRT1 may represent a crucial strategy to prevent age- and obesity-related microvascular dysfunction. Recently, the role of SIRT1 in the mechanism of the increased incidence of deep venous thrombosis (DVT) associated with aging was also investigated recently. Researchers found that DVT was associated with the senescence of endothelial cells and lower SIRT1 expression. The study showed that SIRT1 could counteract endothelial senescence and prevent DVTs. The researchers also discovered that an antisense long non-coding RNA (lncRNA Sirt1-AS) upregulated SIRT1, decreased senescence- and DVT-associated biomarkers in human vascular endothelial cells, and alleviated DVT through upregulating SIRT1. Therefore, lncRNA Sirt1-AS may be a potential new biomarker for DVT (Lou et al., 2021).

In another study by Goto and co-workers investigating the effects of cilostazol on endothelial cell senescence, it was shown that cilostazol increased the expression of SIRT1 in endothelial cells, leading to increased production of NO and decreased expression of cellular senescence markers (Goto, 2005). Overall, SIRT1 plays a crucial role in preserving endothelial function by regulating various cellular pathways that are involved in the development of endothelial dysfunction and has emerged as a potential therapeutic target for the prevention and treatment of cardiovascular diseases.

## 4 SIRT1 and inflammation

Inflammation and coagulation processes are closely interlinked and dependent on each other, with inflammation disrupting the homeostatic balance between pro- and anti-coagulant factors, thereby promoting thrombosis through multiple mechanisms (Becatti et al., 2019a; Emmi et al., 2019a). During inflammation, various cells are activated, producing molecules that contribute the generation of a pro-coagulant state. Inflammatory changes also lead to an increase in procoagulant factors and a decrease in anti-coagulant pathways and fibrinolytic activity, defining the so called “pro-thrombotic diathesis.” Inflammation can cause direct and indirect damage to endothelial cells and inhibit their natural anti-coagulant functions, resulting in a loss of balance in the coagulation system (Di Minno et al., 2012). Studies have shown that inflammation may even directly promote clot formation, and this concept is particularly relevant in venous thromboembolism, where endothelial damage is not always present (Branchford and Carpenter, 2018). The link between inflammation and thrombosis is supported by basic research and clinical epidemiological studies, and clinical trials using anti-inflammatory agents have shown their anti-thrombotic effects (Ridker et al., 2017; Ridker et al., 2018). Among them are statins and anti-interleukin-1 agents, that possess anti-thrombotic and cardioprotective properties beyond or without lipid-lowering effects, likely due to their anti-inflammatory properties which are independent of variations in cholesterol levels (Ridker et al., 2005; Kunutsor et al., 2017; Ridker et al., 2017; Ridker et al., 2018).

In this regard, it has been observed that SIRT1 possesses significant anti-inflammatory properties. For instance, SIRT1 knockdown mice exhibit higher expression of pro-inflammatory cytokines including tumor necrosis factor (TNF) α, IL-1, and IL-6, suggesting a negative association between SIRT1 and inflammation (Yoshizaki et al., 2010). Moreover, the SIRT1 ability to deacetylate NF-κB, a well-known inflammatory mediator, further supports the association between SIRT1 downregulation and thromboinflammation (Lou et al., 2021). In rats with inferior vena cava (IVC) stenosis-induced DVT, SIRT1 protein expression is significantly lower, indicating that DVT is modulated by inflammation via the SIRT1/NF-κB signaling pathway (Lou et al., 2021). This was confirmed in a recent study aimed to investigate the relationship between SIRT1-regulated inflammation and DVT induced by IVC stenosis in rats. Thrombus weight, histopathologic analysis, serum levels of inflammatory cytokines, and protein expressions were evaluated at different time points, and the effects of the SIRT1 activator resveratrol and EX527 (a selective SIRT1 inhibitor) on DVT were assessed. The results suggest that SIRT1 activation attenuated IVC stenosis-induced DVT activating anti-inflammatory pathways (Yao et al., 2019).

Regular exercise is known to have numerous benefits on cardiovascular health, including reducing the risk of thrombosis (Evensen et al., 2018). Obesity and chronic inflammation are associated with an increased risk of blood clots, and regular exercise can help reduce both risk factors. Recently, the effect of resistance exercise on thrombus resolution and recanalization was investigated in a mouse model of DVT. The results showed that resistance exercise did not increase the risk of pulmonary embolism but reduced the weight and size of the thrombus, increased the recanalization rate, and decreased the collagen content. Resistance exercise also inhibited inflammatory responses, increased angiogenesis, and upregulated SIRT1 expression, which may represent the main underlying mechanism of the positive effects of exercise on the thrombotic diathesis (Wu et al., 2023). This is in line with another research aimed at investigate the effects of aerobic exercise after myocardial ischemia/reperfusion injury (MI/RI) in rats with type 2 diabetes mellitus (T2DM). The researchers induced T2DM in male Wistar rats using a high-fat diet and then subjected them to 8 weeks of swimming training. Two days later, all rats underwent MI/RI experiments. The results showed that aerobic exercise decreased serum glucose, total cholesterol, triglycerides, high-density lipoprotein cholesterol, low-density lipoprotein (LDL) cholesterol, and thrombosis in T2DM rats. It also decreased myocardial injury markers and inflammation responses. The study suggests that aerobic exercise can reduce myocardial ischemia/reperfusion injury and repress thrombosis by activating the AMPK/SIRT1/PGC-1α pathway in rats with T2DM (Wang, 2022). Furthermore, SIRT1 plays a key role in suppressing PPAR-𝛾 in mature adipocytes, decreasing fat accumulation. In obesity, an increased number and size of adipocytes are related to a decreased SIRT1 level and activity (Mayoral et al., 2015). Increased adiposity stimulates adipose tissue macrophages to secrete TNF-α, IL-6, and iNOS, leading to inflammation (Makki et al., 2013). Obesity seems to be associated with a lower SIRT1 activity and increased inflammatory response (Vachharajani et al., 2016). Recently Zhou and collaborators performed a study aimed to investigate whether the methoxylated derivative of resveratrol, 3,4′,5-trimethoxy-trans-stilbene (TMS), could alleviate endothelial dysfunction in diabetic and obese mice and its underlying mechanisms. The results showed that the exposure to high glucose levels impaired endothelium-dependent relaxations, decreased NO bioavailability, and downregulated the AMPK/SIRT1/eNOS pathway, while TMS treatment reversed these effects. The protective effects of TMS were mediated by the AMPK/SIRT1/eNOS pathway, and TMS treatment improved endothelial function and attenuated oxidative stress and endoplasmic reticulum stress in aortas of diet-induced obese mice (Zhou et al., 2022). The SIRT1 role in the dysfunction of perivascular adipose tissue (PVAT) induced by a high-fat diet (HFD) was investigated in male C57BL/6J mice. The results showed that the vasodilator response of PVAT-containing aortas to acetylcholine was reduced in obese mice, but the vascular function of PVAT-free aortas remained normal. SIRT1 activity in PVAT of obese mice was lower despite increased SIRT1 expression. The reduced SIRT1 activity was associated with an enhanced acetylation of the eNOS in the PVAT. Additionally, the study found that reduced SIRT1 activity was due to NAD + deficiency, which was likely ascribable to a downregulation of the NAD + -producing enzyme NAMPT. When *ex vivo* PVAT-containing aorta from obese mice were incubated with NAD+, a complete normalization of vascular function was observed. Therefore, an impaired SIRT1 activity due to NAD + deficiency is implicated in obesity-induced PVAT dysfunction (Xia et al., 2022).

The role of SIRT1 in inflammation-induced thrombosis was confirmed by our group in peripheral blood mononuclear cells (PBMC) from giant cell arteritis (GCA) patients. GCA is a condition that affects the elderly and causes inflammation of blood vessels. It can lead to various complications, including venous thrombosis, myocardial infraction, and stroke. The pathogenesis of GCA is not entirely known, but recent studies suggest that oxidative stress, caused by ROS production by immature neutrophils, may play a key role. In this study, 30 GCA patients were compared to 30 healthy controls to evaluate the presence of systemic oxidative stress and alterations in the expression of SIRT1, SIRT6 and SIRT7, sirtuins involved in inhibiting inflammation and oxidative stress. Results showed that GCA patients had significantly higher levels of ROS and plasma oxidative stress markers, such as lipid peroxidation, compared to healthy controls. Additionally, GCA patients had a significant decrease in SIRT1 expression in PBMCs, but not in SIRT6 and SIRT7 expression. The findings suggest that oxidative stress and reduced SIRT1 expression may contribute to GCA pathogenesis (Ianni et al., 2021).

Psoriasis is a chronic autoimmune hyperproliferative cutaneous disease characterized by the presence of sore patches of red, thick skin with silvery scales. Although the link between psoriasis and thrombosis is not fully understood, research has shown that people with psoriasis have a higher risk of developing thrombotic events compared to the general population (Hillary et al., 2021). The underlying mechanisms that contribute to the association between psoriasis and thrombosis are complex and multifactorial. One possible explanation is that inflammation, which is a hallmark of psoriasis, can activate blood clotting factors and increase the risk of thrombosis. Indeed, our study have highlighted an impaired SIRT1 expression and activity in lesional psoriatic fibroblasts, indicating a role of SIRT1 in the pathogenesis of psoriasis (Becatti et al., 2016a). Moreover, psoriatic fibroblasts showed signs of oxidative stress, reduced SIRT1 activity, mitochondrial damage, and increased cell death. However, when SIRT1 was activated, redox balance was restored, mitochondrial function improved, and cell death was reduced (Becatti et al., 2018). The protective role of SIRT1 activation against inflammation and oxidative stress were also confirmed in keratinocytes from vitiligo patients. Vitiligo is a skin disease characterized by depigmented patches on the skin, but its cause is not clear. Our group investigated SIRT1 signaling in perilesional skin biopsies from 16 non-segmental vitiligo patients and found that SIRT1 activation may protect perilesional keratinocytes from damage by downregulating pro-apoptotic molecules [e.g., Jun N-terminal kinase (JNK1) and p38 kinases] and decreasing oxidative stress (Becatti et al., 2014a).

SIRT1 seems therefore a promising avenue for developing novel therapies for inflammation-induced thrombosis, given its critical role in regulating inflammation and its potential as a therapeutic target. However, further research is needed to fully understand the molecular mechanisms of SIRT1 in regulating inflammation and to develop safe and effective SIRT1 activators for clinical use.

## 5 SIRT1 and platelets

Platelets play a critical role in thrombosis, both as essential components of the normal hemostatic response to injury and as key contributors to the pathogenesis of thrombotic disorders when their function is dysregulated or excessive (Koupenova et al., 2017). Studies have shown that SIRT1 levels in platelets can modulate their activation and aggregation, suggesting a role in clot formation (Moscardo et al., 2015). Understanding the mechanisms underlying platelet activation and aggregation is essential for developing effective strategies to prevent and treat thrombosis. In a recent study, the potential neuroprotective effects of prasugrel, a P2Y12 receptor blocker, in cerebral ischemia/reperfusion injury in male Wistar rats was assessed. The study found that prasugrel has anti-ischemic potential beyond its antiplatelet activity by inhibition of the expression of small ubiquitin-related modifier 2/3 (SUMO2/3)-inhibitory kappa (Iκ)Bα, Ubc9, and NF-κB and the activation of cAMP/protein kinase A (PKA)-related pathways, cAMP response element-binding protein (CREB)/Brain Derived Neurotrophic Factor (BDNF), and SIRT1 signaling (Gomaa et al., 2021). The anti-platelet aggregation effects of SIRT1 was also demonstrated by Shen and co-workers examining the anti-platelet aggregation mechanisms of Aspirin eugenol ester (AEE). The study found that AEE significantly inhibited granule secretion markers, intercellular calcium mobilization, and thromboxane B2 formation in agonist-activated platelets *in vitro*. AEE also reduced CD40L activation, suppressed phosphorylation of extracellular signal-regulated kinase 2 (ERK2), c-JNK1, and protein kinase B (AKT), and recovered SIRT1 expression. The study concluded that, *in vitro*, AEE inhibits agonist-induced platelet aggregation via the regulation of SIRT1 pathways (Shen et al., 2019).

Toll-like receptor 4 (TLR4) is a type of receptor protein found on the surface of various cells, including platelets, and is involved in the innate immune response. It has been shown that the activation of platelets by ox-LDL leads to the release of various inflammatory mediators, including cytokines and chemokines, as well as the upregulation of TLR4 expression. TLR4, in turn, activates the NF-κB signaling pathway, leading to the production of pro-inflammatory cytokines such as IL-1β, IL-6, and TNF-α (Rocha et al., 2016). The TLR4-mediated inflammatory response in ox-LDL-activated platelets is implicated in the pathogenesis of atherosclerosis. SIRT1 activation by resveratrol inhibits the TLR4-mediated inflammatory response in ox-LDL-activated platelets, thus likely contributing to the treatment of thrombosis and atherosclerosis. Resveratrol suppressed TLR4 expression, attenuated the ox-LDL-induced phosphorylation of NF-κB and signal transducer and activator of transcription 3 (STAT3), and reduced platelet aggregation and adhesion. This inhibitory effect of resveratrol led to the suppression of AKT phosphorylation, recovering SIRT1 expression and adenosine monophosphate-activated protein kinase phosphorylation, which was reduced by ox-LDL treatment (Sun et al., 2018). The protective role of resveratrol against thrombus formation and platelet aggregation was also confirmed by Kim and colleagues. They found that SIRT1 downregulates platelet-activating factor receptor (PAFR) expression on platelets via proteasomal and lysosomal pathways, decreasing platelet aggregation and pulmonary thrombus formation. SIRT1 activators, such as resveratrol and reSIRT1, were shown to decrease PAFR expression, thus attenuating platelet aggregation and pulmonary thrombus formation (Kim et al., 2016). In this context, the pivotal role of SIRT1 was confirmed by SIRT1 inhibition. Sirtinol and EX-527, inhibitors of SIRT1, induce apoptosis-like modifications in platelets, which lead to increased clearance of the platelets by macrophages. Additionally, sirtinol or EX-527 administration in murine models resulted in a reduced platelet and reticulated platelet count. The study suggests that sirtuins play a central role in determining platelet life span and highlights the potential side effects of sirtuin inhibition in managing thrombosis (Kumari et al., 2015). Overall, these findings suggest that SIRT1 is critical for regulating platelet function and thrombus formation and may represent a promising target for the development of new anti-thrombotic therapies.

## 6 SIRT1 and oxidative stress

It is clearly established that ROS can promote coagulation through several means, such as enhancing the expression of tissue factor in endothelial cells, monocytes, and vascular smooth muscle cells, interfering with platelet activation, and causing oxidative structural and functional alterations to crucial proteins involved in the coagulation cascade (e.g., tissue factor pathway inhibitor (TFPI), protein C, thrombomodulin, fibrinogen, antithrombin) (Gray and Barrowcliffe, 1985; Glaser et al., 1992; Freedman et al., 1999; Van Patten et al., 1999; Ohkura et al., 2004; Nalian and Iakhiaev, 2008; Pensalfini et al., 2008; Barr et al., 2013; Barygina et al., 2013; Becatti et al., 2013; Becatti et al., 2015; Dayal et al., 2015; Becatti et al., 2016b; Becatti et al., 2017; Barygina et al., 2019a; Barygina et al., 2019b; Becatti et al., 2019b; Emmi et al., 2019b; Becatti et al., 2020; Branca et al., 2020; Cito et al., 2020; Ianni et al., 2021; Mannucci et al., 2021; Bettiol et al., 2022; Becatti et al., 2023). Additionally, ROS can facilitate thrombo-inflammation, including via hyperactivation of leukocytes, particularly neutrophils, and the release of neutrophil extracellular traps (NETs) (Bettiol et al., 2021). Also, leukocyte-derived ROS can induce the oxidation of fibrinogen, resulting in an altered secondary structure and overall clot architecture, displaying reduced porosity and a tight fibrin network with filaments of reduced average size (Miniati et al., 2010; Becatti et al., 2014b; Becatti et al., 2016c; Becatti et al., 2020). These oxidative alterations lead to dysfunctional fibrinogen features, both in terms of thrombin-catalyzed fibrin polymerization and susceptibility to plasmin-induced lysis (Pensalfini et al., 2008; Miniati et al., 2010; Barygina et al., 2013; Becatti et al., 2013; Cellai et al., 2013; Becatti et al., 2014b; Cellai et al., 2014; Lami et al., 2014; Becatti et al., 2015; Becatti et al., 2016b; Becatti et al., 2016c; Becatti et al., 2017; Barygina et al., 2019a; Barygina et al., 2019b; Becatti et al., 2019b; Emmi et al., 2019b; Becatti et al., 2020; Branca et al., 2020; Cito et al., 2020; Ianni et al., 2021; Mannucci et al., 2021; Bettiol et al., 2022; Becatti et al., 2023).

As previously described, endothelium-specific overexpression of SIRT1 upregulates eNOS expression, which enhances vascular endothelium-dependent vasodilation (Man et al., 2019). Endogenous NO is known to be an anti-atherosclerotic and anti-aging factor, and SIRT1 in endothelial cells can modulate NO production. Age-associated endothelial dysfunction in mice has been linked to lower SIRT1 expression and eNOS activity (Begum et al., 2021).

Mattagajasingh and collaborators found that SIRT1 induces endothelium vasodilation by activating eNOS and increasing NO production in calorie-restricted mice (Mattagajasingh et al., 2007). SIRT1 role in regulating eNOS and oxidative stress is crucial for understanding its role in thrombosis and atherosclerosis pathways. SIRT1 has been identified as an antioxidative stress molecule and has been studied for its role in ROS resistance in endothelial cells. Studies have proposed that SIRT1 improves endothelium-dependent vascular function by activating eNOS and reducing oxidative stress. Laminar shear stress can promote SIRT1 activity and activate eNOS. Resveratrol-induced SIRT1 activation suppresses angiotensin II type I receptor expression in vascular smooth muscle cells, which prevents vessel contraction and blood pressure increase (Miyazaki et al., 2008). SIRT1 deacetylates and activates eNOS *in vitro*, promoting vascular function in aortic rings *ex vivo*. A temporary increase in ROS can stimulate SIRT1, which in turn causes a decrease in ROS, even though high ROS levels are associated with lower SIRT1 activation. This may be related to the involvement of different cellular activities in preventing ROS damage. There is a complex signaling network for SIRT1-mediated ROS reduction involving SIRT1/FoxOs, SIRT1/NF-κB, SIRT1/NOX, SIRT1/SOD, and SIRT1/eNOS. Among these factors, eNOS, SOD, and FoxO can also promote SIRT1 activation via a positive feedback mechanism (Zhang et al., 2017a).

The positive interactions among various antioxidant agents can enhance their protective effect against oxidative stress. The protective role of SIRT1 against oxidative stress and aging was demonstrated in a study aiming to investigate the role of oxidative stress in regulating lifespan in living organisms. The study showed that glutathione (GSH), a free radical scavenger system, can help maintain redox status and act as an antioxidant. The study also introduced a series of novel S-acyl-GSH derivatives, which can reduce oxidative stress and cholinergic dysfunction in models of Alzheimer’s disease. The study demonstrated that linolenoyl-SG (lin-SG) thioester, when taken as a dietary supplement, can significantly increase the lifespan of the wild-type N2 *Caenorhabditis elegans* strain compared to other supplements like the ethyl ester of GSH, linolenic acid, or vitamin E. The study suggested that the increase in lifespan is due to the upregulation of Sir-2.1, an NAD-dependent histone deacetylase ortholog of mammalian SIRT1. The study also showed that overexpression of Sir-2.1 mediated by lin-SG is related to the Daf-16 (FoxO) pathway. Furthermore, the lin-SG derivative protected N2 worms from paralysis and oxidative stress induced by Aβ and H2O2 exposure. These findings suggested that lin-SG thioester can be used as an antioxidant supplement to trigger sirtuin upregulation, with a promising role against aging and oxidative-related diseases (Cascella et al., 2014).

NF-κB-mediated suppression of SIRT1 activation, which is associated with increased levels of ROS and reduced ROS scavenging, underscores the significance of SIRT1 as a key target of NF-κB (Wan and Garg, 2021).

The promoter sequences of Sirt1 gene have a number of putative binding sites for the NF-κB transcription factor (Voelter-Mahlknecht and Mahlknecht, 2006), but the role of NF-κB in the control of Sirt1 transactivation is still largely unclear. NF-κB signaling seems to downregulate SIRT1 via the increase in the expression of miR-34a (Li et al., 2012a), a miRNA involved in the control of immune and metabolic events and whose levels are known to increase with aging in mouse brain and blood cells. Moreover, NF-κB signaling stimulates the expression of IFN-γ, which suppresses the SIRT1 by increased the expression of CIITA and HIC1, a well-known inhibitor of SIRT1 transcription, and reducing the expression of SIRT1 at the transcriptional level (Li et al., 2012b). Also, NF-κB signaling can downregulate SIRT1 by inducing the protein components of NADPH oxidase complex (gp91phox and p22phox), thereby increasing ROS levels which can directly oxidize the cysteine residues of SIRT1, inactivating it and targeting it towards degradation in proteasomes (Anrather et al., 2006; Rajendrasozhan et al., 2008). Concomitantly, ROS also reduces the cellular level of NAD+, an obligatory substrate for SIRT1 activity (Du et al., 2003).

The interplay between NOX inhibition and NF-κB suppression in endothelial cells highlights the interaction between NOX and NF-κB signaling (Park et al., 2016), which has been implicated in endothelial dysfunction caused by high-dose intravenous iron supplementation. Furthermore, NOX2-p47phox complex-mediated eNOS phosphorylation and NO production in ECs exposed to laminar shear stress, and the uncoupling of eNOS by NOX1-NOXO1 under atherogenic oscillatory shear stress can lead to endothelial cell injury (Ungvari et al., 2018).

SIRT1-dependent suppression of the NF-κB-mediated inflammatory response in human monocyte cells exposed to cigarette smoke extracts highlights the role of SIRT1 in mitigating ROS-mediated inflammatory pathways (Hwang et al., 2013). The increased neutrophil ROS production in patients with GCA compared to healthy subjects, which can result in higher protein oxidation and permeability of the endothelial barrier *in vitro*, underscores the contribution of immature neutrophil-derived ROS production to the pathogenesis of vascular complications in GCA (Ianni et al., 2021). Moreover, in ventricular cardiomyocytes of neonatal rats subjected to simulated ischemia-reperfusion injury, SIRT1 overexpression induced by resveratrol had a protected role against oxidative injury, mitochondrial dysfunction, and cell death. The study also found that SIRT1 overexpression positively affects the mitogen-activated protein kinase (MAPK) pathway, via Akt/ASK1 signaling, by reducing p38 and JNK phosphorylation and increasing ERK phosphorylation (Becatti et al., 2012). More recently, the protective role of SIRT1 was confirmed in a study aiming at assessing the mechanisms sustaining the beneficial effects of ticagrelor compared to clopidogrel in patients with stable coronary artery disease and chronic obstructive pulmonary disease undergoing percutaneous coronary intervention. The researchers analyzed RNA levels of markers of inflammation and oxidative stress in peripheral blood cells and found that ticagrelor led to enhanced levels of SIRT1 and hairy and enhancer of split-1 (HES1) mRNA, two genes involved in anti-inflammatory and anti-oxidant mechanisms. This suggests that ticagrelor’s positive effects on biological markers of endothelial function may be mediated by its ability to counteract systemic inflammation and oxidative stress (Aquila et al., 2020).

## 7 SIRT1 and lipid metabolism

SIRT1 can regulate lipid metabolism and protect against atherosclerosis. SIRT1 suppresses foam cell formation in atherosclerotic plaques by enhancing the LXR- ATP Binding Cassette Subfamily A Member 1 (ABCA1)/ATP binding cassette subfamily G member 1 (ABCG1)/chemokine (C-C motif) receptor 7 (CCR7) pathway, as well as stopping the expression of scavenger receptor Lox-1 in macrophages, thus reducing the uptake of oxLDL and preventing macrophage foam cell formation (Xu et al., 2019).

In the inflammatory context, SIRT1 reduces the expression of proinflammatory molecules and modulates insulin sensitivity (Kitada et al., 2019). SIRT1 activation also weakens hepatic secretion of the serine protease, proprotein convertase subtilisin/kexin type 9 (PCSK9), which promotes lysosomal degradation of hepatic low-density lipoprotein receptor (LDLR) and counteracts its recycle to the cell surface. The accumulation of PCSK9 increases LDLR protein degradation and enhances the plasma clearance of LDL-cholesterol, leading to decreased plaque formation (Lagace, 2014).

Furthermore, SIRT1 promptly deacetylates and regulates the transcriptional activity of LXR, a crucial regulator of lipid homeostasis and inflammation. Activation of LXRα is associated with the expression of ABCA1, which regulates the efflux of cholesterol into pre-βHDL particles. Thus, cholesterol efflux from SIRT1^+/+^ peritoneal macrophages is higher than from SIRT1−/− peritoneal macrophages *ex vivo* (Feng et al., 2018).

The key role of SIRT1 in atherosclerosis is now established. In 2010, SIRT1 was emerged as a crucial player in the regulation of tissue metalloproteinase 3 (TIMP3), an endogenous enzyme with a role in vascular inflammation and preventing atherosclerosis. Authors showed that at level of human carotid atherosclerotic plaques, TIMP3 was significantly impaired in patients with type 2 diabetes, leading to A Disintegrin And Metalloproteinase (ADAM17) and MMP9 overactivity. Reduced expression of TIMP3 was associated *in vivo* with SIRT1 levels. In smooth muscle cells, a reduction of SIRT1 activity and levels decreased TIMP3 expression, while SIRT1 overexpression increased TIMP3 promoter activity. This study suggested that in patients with type 2 diabetes, the deregulation of ADAM17 and MMP9 activities in atherosclerotic plaques is associated with an impaired expression of TIMP3 via SIRT1 (Cardellini et al., 2009).

The rupture of an atherosclerotic plaque is a well-known trigger of thrombosis, which is related to highly thrombogenic material that induces platelet recruitment and aggregation and increases the size of the new thrombus. Tissue factor, a coagulation factor, is significantly expressed in atherosclerotic plaques and released into the blood during plaque rupture, activating the coagulation and platelets. Many studies suggest that SIRT1 may prevent atherothrombosis by downregulating endothelial expression of tissue factor. Barbieri and co-workers demonstrated that the deletion of cyclooxygenase (COX) 2 decreased the production of prostacyclin synthase, peroxisome proliferator-activated receptor, and SIRT1, leading to increased upregulation of tissue factor. They discovered that modulation of SIRT1 and tissue factor by prostacyclin/peroxisome proliferator-activated receptor-δ pathways can control arterial thrombus formation, which could represent a therapeutic target against atherothrombotic complications associated with COX-2 inhibitors (Barbieri et al., 2012). Moreover, *in vitro* experiments using human and mouse cell lines, and *in vivo* experiments using C57Bl/6 mice, showed that inhibition of SIRT1 increased cytokine-induced endothelial tissue factor expression and activity. Tissue factor mRNA expression, tissue factor promoter activity, and nuclear translocation were also enhanced, along with the DNA binding of the p65 subunit of NFκB. Mice treated with the SIRT1 inhibitor showed an increased tissue factor activity in the arterial vessel wall and carotid artery thrombus formation (Breitenstein et al., 2011). More recently, the interplay between SIRT1 and tissue factor was confirmed in a study aimed to investigate the effect of chronic exposure to indoxyl sulfate on the hemostatic system and arterial thrombosis in a rat model. Chronic exposure to indoxyl sulfate was found to promote arterial thrombosis by increasing the levels of complex TF/factor VII, plasminogen activator inhibitor-1 (PAI-1), and platelet activation, as well as by decreasing aortic levels of SIRT1, thus further supporting the protective role of SIRT1 in thrombosis (Karbowska et al., 2018).

The anti-atherosclerotic effect of SIRT1 was also confirmed in another study where it has been found that evogliptin inhibited the activation of the transcription factor NF-κB induced by TNF-α, which was mediated by the interaction of NF-κB with SIRT1. The study suggested that the interaction between NF-κB and SIRT1 may be a potential target for the development of anti-atherosclerotic therapies. Additionally, the study has been demonstrated that the SIRT1 gene knockdown reversed the inhibitory effect of evogliptin on TNF-α-mediated adhesion between endothelial cells and monocytes, indicating that SIRT1 is involved in the mechanism of action of evogliptin in inhibiting arterial inflammation (Nguyen et al., 2019). Similar results were obtained by Quian and collaborators in a study aimed to investigated the potential of G004, a synthetic sulfonylurea compound, in suppressing the onset and development of atherosclerosis in mice. The study showed that G004 improved serum lipid accumulation, atherosclerotic lesions, and liver steatosis. It also increased the expression of SIRT1 and eNOS, promoting reverse cholesterol transport and alleviating inflammation. The study concluded that G004 could be an effective anti-atherosclerosis agent stimulating SIRT1/eNOS (Qian et al., 2017).

Vascular inflammation-mediated atherosclerosis was also investigated by Raj and collaborators. They explored the effect of a combination treatment with metformin and cholecalciferol on lipopolysaccharide (LPS)-induced endothelial cell activation. LPS exposure leads to increased biosynthesis of pro-inflammatory mediators, cellular injury, and vascular inflammation. The study found that the dual compound treatment inhibited LPS-induced protein arginine methylation, endothelial senescence, and dysfunction through SIRT1 pathway (Raj et al., 2021). The role of SIRT1 in arterial thrombus formation was explored by Akhmedov and co-workers in a study aimed to investigate the role of lectin-like oxLDL receptor-1 (LOX-1) in arterial thrombus formation. LOX-1 promotes endothelial uptake of oxLDL and is known to play a role in atherosclerosis and acute coronary syndrome (ACS). The researchers used endothelial-specific LOX-1 transgenic mice and a photochemical injury thrombosis model of the carotid artery to examine the effect of LOX-1 on thrombus formation at different levels of oxLDL. The results showed that LOX-1 differentially regulated thrombus formation *in vivo* depending on the level of oxLDL. At low oxLDL levels, LOX-1 activated the protective SIRT1 pathway, which reduced tissue factor (TF) expression and activity, leading to prolonged occlusion time. However, at higher levels of oxLDL, LOX-1 switched to the thrombogenic ERK1/2 pathway, resulting in an enhanced thrombotic response and shortened occlusion time. The findings suggested that SIRT1 may represent a novel therapeutic target for arterial thrombus formation in ACS (Akhmedov et al., 2017). The role of SIRT1 activation on lipid metabolism regulation was confirmed by a study that investigated the effects of a naphthofuran derivative BF4, a potent SIRT1 activator, on lipid metabolism in 3T3-L1 adipocytes. The results indicated that BF4 significantly decreased adipogenesis and lipid accumulation and inhibited the differentiation of 3T3-L1 pre-adipocytes into adipocytes. BF4 also decreased the expressions of several key regulators in adipocyte differentiation, including C/EBPβ and PPARγ, and their downstream lipogenesis targets via the activation of the SIRT1 pathway (Gao et al., 2023). The development of atherosclerosis is also related to mithocondrial dysfunction and oxidative stress. Karnewar and collaborators, synthesized a mithocondrial-targeted antioxidant called Mito-Esc, an alkyl TPP + -tagged esculetin to investigate its efficacy in mitigate atherosclerosis in Apoe−/− mice. Chronic low-dose administration of Mito-Esc prevented alterations in lipid profile, blood pressure and atherosclerotic plaque formation. Mito-Esc also improved mitochondrial function and delayed endothelial cell senescence by increasing human telomerase reverse transcriptase (hTERT) levels via SIRT1 activation (Karnewar et al., 2022). Moreover, it has been suggested that SIRT1 activation inhibits iron-induced ferroptosis in foam cells and decreases IL-1β and IL-18 levels by restoring autophagy flux (Su et al., 2021). The key role of SIRT1 in lipid metabolism was confirmed by Bruckbauer and collaborators in a study that investigated the effects of combining leucine and nicotinic acid (a lipid-lowering drug) on AMPK/SIRT1 signaling and lipid metabolism in cell culture, *C. elegans*, and mice. The results showed that the combination of leucine and low dose nicotinic acid increased AMPK and SIRT1 in adipocytes and myotubes, reduced lipid accumulation and increased median survival in *C. elegans*, and improved lipid metabolism, hyperlipidemia, and atherosclerosis in mice, suggesting that the combination of leucine and nicotinic acid may be a potential alternative therapy for hyperlipidemia and atherosclerosis by activating the AMPK/SIRT1 axis (Bruckbauer et al., 2017).

Overall, these findings suggest that SIRT1 plays a crucial role in regulating lipid metabolism and inflammation in the context of atherosclerosis, making it a potential therapeutic target for the prevention and treatment of this disease.

## 8 Natural compounds as SIRT1 modulators

As mentioned in the above paragraphs, natural compounds exist, such as resveratrol, displaying sirtuin-modulating actions. Indeed, natural products have an intrinsically high ability to bind biomacromolecules, with a great potential for the discovery of new therapeutic scaffolds and for the subsequent development of isoform selective, potent, and cell-permeable compounds with suitable pharmacokinetic properties for clinical use (Table 1).

**TABLE 1:** Main SIRT1 signalling pathways targeted by SIRT1 modulators tested in preclinical or clinical studies.

Deacetylation targets	Biological pathways	SIRT1 modulators	Models in which the modulator was tested
AMPK/LXR	Hyperlipidemia and atherosclerosis; mitochondrial biogenesis	-Resveratrol Price et al., (2012)	-Mice Price et al., (2012)
		-Quercetin Hung et al., (2015); Qiu and Luo, (2018)	-In vitro Hung et al., (2015) and in osteoarthritis rats Qiu and Luo, (2018)
		- Curcumin Lin et al., (2015)	- Curcumin Lin et al., (2015)
		-Leucine and nicotinic acid Karnewar et al., (2022)	-In vitro, *C. elegans*, and mice Karnewar et al., (2022)
AP-1	Endothelial activation in dyslipidemia	-Curcumin Han et al., (2002)	- Human promyelocytic leukemia cells Han et al., (2002)
eNOS	Endothelial function	-Cilostazol Ota et al., (2008); Wu et al., (2021a)	- *In vitro* (HUVEC) Ota et al., (2008); Wu et al., (2021a)
		-G004 Karbowska et al., (2018)	-Mice Karbowska et al., (2018)
FoxO	Apoptosis, authophagy, oxidative stress vWF and P-selectin release, sense nutrient availability	-Resveratrol Li et al., (2020)	-Subtotal nephrectomy rats Li et al., (2020)
		-Berberine Yu et al., (2016)	-Myocardial ischemia/reperfusion rats Yu et al., (2016)
		-Linolenoyl-SG (lin-SG) thioester Cascella et al., (2014)	-N2 *Caenorhabditis elegans* Cascella et al., (2014)
		-Fisetin Kim et al., 2015)	-3T3-L1 cells Kim et al., 2015)
FXR	Metabolic regulation, inflammation, cell death, fibrogenesis	- SRT1720 Cui et al., (2023)	-Mice Cui et al., (2023)
hTERT	Mitochondrial function	Mito-Esc Akhmedov et al., (2017)	Apoe (−/−) mice Akhmedov et al., (2017)
JNK1	Apoptosis, platelet aggregation	Aspirin eugenol ester Shen et al., (2019)	*In vitro* and *ex vivo* (rats) Shen et al., (2019)
MnSOD	Oxidative stress	-Resveratrol Li et al., (2020)	Subtotal nephrectomy rats Li et al., (2020)
		-Curcumin Seshadri et al., (2015)	- Mouse embryonic stem cell Seshadri et al., (2015)
NF-κB	Oxidative stress/thrombo-inflammation	-Resveratrol Yeung et al., (2004); Han et al., (2020)	- *In vitro* Yeung et al., (2004); Han et al., (2020)
		-Quercetin Iskender et al., (2017)	- Diabetic rats Iskender et al., (2017)
		- Hesperidin Iskender et al., (2017)	- Diabetic rats Iskender et al., (2017)
		- Curcumin Chuang et al., (2002); Han et al., (2002)	-Human carcinoma cells; human promyelocytic leukemia cells Chuang et al., (2002); Han et al., (2002)
		-Evogliptin Breitenstein et al., (2011)	-Mice Breitenstein et al., (2011)
p53	Apoptosis	-YK-3-237 Yi et al., (2013)	- Breast cancer cells Yi et al., (2013)
		-Curcumin Zhang et al., (2017b)	- Albino stroke‐induced model rats Zhang et al., (2017b)
p66Shc and Arginase II	Microvascular dysfunction, DVT	-SRT1720 Chen et al., (2020)	- *Ex Vivo* Human Vessels Chen et al., (2020)
		-lncRNA Sirt1-AS Luo et al., (2014)	-In vitro (HUVEC) Luo et al., (2014)
PAFR	Platelet aggregation	- Resveratrol, reSIRT1 Kim et al., (2016)	- *In vitro* (isolated human platelets) Kim et al., (2016)
PGC-1α	Mitochondrial biogenesis; apoptosis	-Quercitin Tang et al., (2019)	- *In vitro* and in rats (ischemia/reperfusion models) Tang et al., (2019)
		- Curcumin Lagouge et al., (2006)	- Epididymal white adipose murine tissue Lagouge et al., (2006)
PPARδ	Endothelial function	Resveratrol Cheang et al., (2019)	PPARδ mutant obese mice Cheang et al., (2019)
PPARγ	Adipocyte differentiation and maturation	−3,4′,5-trimethoxy-trans-stilbene (TMS) Zhou et al., (2022)	-Diabetic/obese mice Zhou et al., (2022)
		- Naphthofuran derivative BF4 Raj et al., (2021)	-3-T3-L1 adipocytes Raj et al., (2021)
		-Fisetin Kim et al., (2015)	- 3T3-L1 cells Kim et al., (2015)
SOD1	Oxidative stress	- Berberine (Chen and Yang, (2017)	-In vitro and in diabetic mouse model Chen and Yang, (2017)
SREBP	Lipid and sterol homeostasis	- SRT1720 Walker et al., (2010)	- Obese mice Walker et al., (2010)

AMPK: 5′ adenosine monophosphate-activated protein kinase; AP-1: activator protein-1; eNOS: endothelial nitric oxide synthase; FoxO: forkhead transcription factor O; FXR: farnesoid X receptor; hTERT: human telomerase reverse transcriptase; HUVEC: Human umbilical vein endothelial cells; JKN: Jun N-terminal kinase; LXR: liver X receptors; MnSOD: manganese-dependent superoxide dismutase; NF-κB: nuclear factor κB; PAFR: platelet-activating factor receptor; PGC-1α: peroxisome proliferator-activated receptor-γ co-activator-1α; PPAR: Peroxisome proliferator-activated receptor; SREBP: sterol regulatory element binding protein; vWF: von Willebrand factor.

Using high-throughput or in silico-based screening or fragment-based drug discovery approaches, several molecules displaying activation or inhibition actions against sirtuins have been identified.

Among them are stilbenoids, including resveratrol, piceatannol, and trans-(−)-ϵ-viniferin. Resveratrol is a natural polyphenol, present in high amounts in plants such as grapes, cranberries, blueberries, peanuts, groundnuts and Japanese knotwee (Burns et al., 2002). It is a weak inhibitor of SIRT2 and SIRT3, and a stimulator of SIRT1 and SIRT5 activity, and its action on SIRT1 is mediated by AMPK (Gertz et al., 2012). An X-ray crystal structure of SIRT1 in complex with three resveratrol molecules and a 7-amino-4-methylcoumarin (AMC)-containing peptide could be solved, and structural and functional analysis indicated that resveratrol could only stimulate SIRT1 in the presence of an N-terminal domain in addition to the catalytic domain (Cao et al., 2015). Due to its low bioavailability and solubility as a natural compound, a different resveratrol formulation (SRT501) was developed by Sirtris Pharmaceuticals (Cambridge, MA, United States). However, serious renal adverse effects led to the precocious interruption of a Phase IIa study comparing SRT501 monotherapy vs. SRT501 + bortezomib for advanced multiple myeloma (Villalba et al., 2012). Recent studies providing insights into the binding sites and the mechanisms of action of resveratrol-like compounds might pave the way for the development of resveratrol analogs with higher potency and selectivity for specific sirtuin isoforms.

A resveratrol metabolite, piceatannol, could also activate SIRT1 and inhibit SIRT3, whereas viniferin was shown to increase mitochondrial SIRT3 levels and indirectly activate SIRT3-dependent deacetylation (Fu et al., 2012).

Regarding chromone-derived compounds, phytoestrogenic isoflavones (including daidzein and genistein) which are present in large amounts in soy products, display SIRT1 activating actions, resulting in inhibitory effects on muscle atrophy in C2C12 myotubes (Wen et al., 2018).

The flavonoid polyphenol quercetin is a natural dietary supplement present in fruits, vegetables, and nuts, and it is known for its antioxidant and anti-inflammatory properties, as well as for its antiproliferative, chemopreventive and anticarcinogenic activities (Iside et al., 2020). Quercetin can modulate various pathways involving SIRT1, including AMPK/LXR axis (Qiu and Luo, 2018), NF-κB signalling (Iskender et al., 2017), PGC-1α (Tang et al., 2019). Accordingly, evidence coming from a number of clinical trials demonstrated a benefic effect of quercitin for different conditions, such as T2D (NCT01839344), mucositis (NCT01732393), hepatitis C (NCT01438320), idiopathic pulmonary fibrosis (NCT02874989), osteoporosis (NCT00330096), uric acid metabolism (NCT01881919), cytokine release (NCT01106170), and chronic obstructive pulmonary disease (NCT01708278).

Similarly, curcumin is a natural bioactive polyphenol compound mediating antioxidant, anticancer, and anti-inflammatory properties (Iside et al., 2020). Via SIRT1 modulation, curcumin can interfere with the AMPK/LXR axis (Lin et al., 2015), as well as with AP-1 (Han et al., 2002), manganese-dependent SOD (MnSOD) (Seshadri et al., 2015), NF-κB (Chuang et al., 2002), p53 (Zhang et al., 2017b), and PGC-1α (Lagouge et al., 2006). Various completed and terminated clinical trials assessed the effects of curcumin in metabolic and inflammatory diseases, including in subjects at risk for metabolic syndrome (NCT01925547), in inflammation induced by endometrial carcinoma (NCT02017353) and by spinal cord injury (NCT02099890), and in preventing or reducing radiation-induced dermatitis in breast cancer patients receiving radiotherapy (NCT01246973, NCT01042938, NCT02556632).

As for the dietary flavonoid fisetin, it is a natural polyphenol that can be found in plants and fruits such as apples, persimmons, grapes, onions, kiwi, kale, and strawberries (Iside et al., 2020). It was shown to modulate the SIRT1/PPARγ pathway (Kim et al., 2015) as well as the SIRT1/FoxO axis (Kim et al., 2015), and its anti-inflammatory effects have been tested in clinical trials on Frail Elderly Syndrome (NCT03675724 and NCT03430037), symptomatic knee osteoarthritis patients (NCT04210986), chronic kidney diseases and diabetes (NCT03325322).

Berberine is another natural phytocompound; it is an isoquinoline alkaloid displaying analgesic, anticancer, anti-inflammatory, and myocardial protective properties (Iside et al., 2020). These actions are mediated also by its interference with different SIRT1 pathways, including the FoxO (Yu et al., 2016) and the SOD1 axis (Chen and Yang, 2017). These promising results paved the way to clinical trials on berberine for different conditions, including diabetes and dyslipidemia (NCT00425009 and NCT00462046), hyperlipidemia and hypercholesterolemia (including NCT02422927, NCT03216811 and NCT03470376).

Conversely, synthetic stereoisomers of the tanikolide dimer from the Madagascan Marine Cyanobacterium *Lyngbya majuscula* and bichalcones from the African Medicinal Plant Lyngbya majuscula showed moderate inhibitory activities against SIRT1. Particularly, 3,2′,3′,4′-tetrahydroxychalcone display a stronger inhibitory effect on the SIRT1-pathway than the known SIRT1-inhibitor sirtinol (Singh et al., 1999; Kahyo et al., 2008).

## 9 Conclusion

A growing body of evidence suggests that SIRT1 has a broad range of effects on the regulation of numerous cellular pathways, including inflammatory, procoagulant and atherosclerotic ones. Numerous preclinical and clinical studies have demonstrated that SIRT1 possesses anti-inflammatory and anti-thrombotic properties, exerting a protective role against atherosclerosis and plaque rupture, platelet activation, oxidative-stress induced thrombosis, as well as adiposity- and age-related vascular dysfunction.

Overall, SIRT1 activation seems a promising strategy for preventing inflammation-induced thrombosis, and pharmacological sirtuins-activating compounds such as resveratrol have shown anti-inflammatory, anticoagulatory, and anti-fibrinolytic effects *in vitro*. Moreover, artificial SIRT1 activators, such as SRT2104, are currently under clinical investigation, with future potential therapeutic applications in the field of cardiovascular and metabolic diseases.
