# Supplementary material for: SIRT1 and thrombosis
Source: Front Mol Biosci. 2024 Jan 18;10:1325002. doi: 10.3389/fmolb.2023.1325002 (PMC10833004; doi:10.3389/fmolb.2023.1325002)
Supplement: Supplementary file 1 [file DataSheet1.pdf]

CLUSTAL O(1.2.4) multiple sequence alignment

```
sp|Q96EB6|SIR1_HUMAN      MA-DEAALALQPGGSPSAAGADREAASSPAGEPLRKRPRRDGPGLERSPGEPGGAAPERE  59
sp|P06700|SIR2_YEAST      MTIPHMKYAVSKTS---ENKVSNTVSPTQDKDAIRKQPDDI-----INNDEPSHKKIKVA  52
                          *:  .   *:.  .      ... .: :   : :**:*         . .**.    :

sp|Q96EB6|SIR1_HUMAN      VPAAARG-----CPGAAAAALWREAEAEAAAAGGEQEAQAT-----------AAAGEGDN  103
sp|P06700|SIR2_YEAST      QPDSLRETNTTDPLGHTKAALGEVASMELKPT-NDMDPLAVSAASVVSMSNDVLKPETPK  111
                           * : *        * : *** . *. *   : .: :  *.           .   *  :

sp|Q96EB6|SIR1_HUMAN      GPGLQGPSREPPLADNLYDEDDDDEGEEEEEAAAAAIGYRDNLLFGDEIITNGFHSCESD  163
sp|P06700|SIR2_YEAST      GPIIISK---NPSNGIFYGPSFTK---RESLNARMFLKYYGAHKF---------------  150
                          ** : .     *  . :*. .  .   .*.  *   : * .   *

sp|Q96EB6|SIR1_HUMAN      EEDRASHASSSDWTPRPRIGPYTFVQQ---------HLMIGTDPRT-------ILKDLLP  207
sp|P06700|SIR2_YEAST      ---------LDTYLPEDLNSLYIYYLIKLLGFEVKDQALIGTINSIVHINSQERVQDLG-  200
                                    . : *.   . * :            : :***            ::**

sp|Q96EB6|SIR1_HUMAN      ETIPPPELDDMTLWQIVINI--------LSEPPKRKKRKDINTIEDAVKLLQECKKIIVL  259
sp|P06700|SIR2_YEAST      SAISVTNVEDPLAKKQTVRLIKDLQRAINKVLCTRLRLSNFFTIDHFIQKLHTARKILVL  260
                          .:*   :::*    : .:.:         .   .* : .:: **:. :: *: .:**:**

sp|Q96EB6|SIR1_HUMAN      TGAGVSVSCGIPDFRSRDGIYARLAVDFPDLPDPQAMFDIEYFRKDPRPFFKFAKEIYPG  319
sp|P06700|SIR2_YEAST      TGAGVSTSLGIPDFRSSEGFYSKIK--HLGLDDPQDVFNYNIFMHDPSVFYNIANMVLPP  318
                          ******.* ******* :*:*:::   . .* *** :*: : * :**  *:::*: : *

sp|Q96EB6|SIR1_HUMAN      QFQPSLCHKFIALSDKEGKLLRNYTQNIDTLEQVAGIQ--RIIQCHGSFATASCLICKYK  377
sp|P06700|SIR2_YEAST      EKIYSPLHSFIKMLQMKGKLLRNYTQNIDNLESYAGISTDKLVQCHGSFATATCVTCHWN  378
                          :   *  *.** : : :***********.**. ***.  :::*********:*: *:::

sp|Q96EB6|SIR1_HUMAN      VDCEAVRGDIFNQVVPRCPRCPADE------------------------------PLAIM  407
sp|P06700|SIR2_YEAST      LPGERIFNKIRNLELPLCPYCYKKRREYFPEGYNNKVGVAASQGSMSERPPYILNSYGVL  438
                          :  * : ..* *  :* ** *  ..                                .::

sp|Q96EB6|SIR1_HUMAN      KPEIVFFGENLPEQFHRAMKYDKDEVDLLIVIGSSLKVRPVALIPSSIPHEVPQILINRE  467
sp|P06700|SIR2_YEAST      KPDITFFGEALPNKFHKSIREDILECDLLICIGTSLKVAPVSEIVNMVPSHVPQVLINRD  498
                          **:*.**** **::**:::: *  * **** **:**** **: * . :* .***:****:

sp|Q96EB6|SIR1_HUMAN      PLPHLHFDVELLGDCDVIINELCHRLGGEYAKLCCNPVKLSEITEKPPRTQKELAYLS--  525
sp|P06700|SIR2_YEAST      PVKHAEFDLSLLGYCDDIAAMVAQKCGWTIPHKKWNDLKNKNFKCQE---KDKGVYVVTS  555
                          *: * .**:.*** ** *   :.:: *    :   * :* .::. :    :.: .*:

sp|Q96EB6|SIR1_HUMAN      ELPPTPLHVSEDSSSPERTSPPDSSVIVTLLDQAAKSNDDLDVSESKGCMEEKPQEVQTS  585
sp|P06700|SIR2_YEAST      DEHPKTL-----------------------------------------------------  562
                          :  *. *

sp|Q96EB6|SIR1_HUMAN      RNVESIAEQMENPDLKNVGSSTGEKNERTSVAGTVRKCWPNRVAKEQISRRLDGNQYLFL  645
sp|P06700|SIR2_YEAST      ------------------------------------------------------------  562


sp|Q96EB6|SIR1_HUMAN      PPNRYIFHGAEVYSDSEDDVLSSSSCGSNSDSGTCQSPSLEEPMEDESEIEEFYNGLEDE  705
sp|P06700|SIR2_YEAST      ------------------------------------------------------------  562


sp|Q96EB6|SIR1_HUMAN      PDVPERAGGAGFGTDGDDQEAINEAISVKQEVTDMNYPSNKS    747
sp|P06700|SIR2_YEAST      ------------------------------------------    562
```

```
#
#
#  Percent Identity  Matrix - created by Clustal2.1
#
#

     1: sp|Q96EB6|SIR1_HUMAN  100.00   32.44
     2: sp|P06700|SIR2_YEAST   32.44  100.00
```

https://www.ebi.ac.uk/Tools/services/rest/clustalo/result/clustalo-R20231215-144932-0261-73232030-p1m/pim[15/12/2023 15:57:45]
